# Mucosal DNA methylome alteration in Crohn’s disease: surgical and non-surgical groups

**DOI:** 10.3389/fgene.2023.1244513

**Published:** 2023-11-17

**Authors:** Saeed Ahmad, Mia Sands, Eugene Greenberg, Lyn Tangen, Jiacheng Huang, Joseph Maria Kumar Irudayaraj

**Affiliations:** ^1^ Biomedical Research Center, Mills Breast Cancer Institute, Carle Foundation Hospital, Urbana, IL, United States; ^2^ Department of Bioengineering, University of Illinois Urbana-Champaign, Champaign, IL, United States; ^3^ Digestive Health Institute, Carle Foundation Hospital, Urbana, IL, United States; ^4^ Carl Woese Institute for Genomic Biology, University of Illinois Urbana-Champaign, Champaign, IL, United States; ^5^ Cancer Center at Illinois, University of Illinois Urbana-Champaign, Champaign, IL, United States; ^6^ Carle Illinois College of Medicine, University of Illinois Urbana-Champaign, Champaign, IL, United States

**Keywords:** Crohn’s disease, epigenome, DNA methylation, surgical/non-surgical patients, reduced representation bisulfite sequencing

## Abstract

Crohn’s disease (CD) is characterized as a chronic, relapsing, and progressive disorder with a complex etiology involving interactions between host, microbiome, and the external environment. Genome wide association studies (GWAS) suggest several genetic variations in the diseased individuals but that explains only a small proportion of susceptibility to disease conditions. This indicates the possible role of epigenome which links environmental factors to the genetic variation in the disease etiology. The current study is focused on the DNA methylome evolution with disease progression. We performed Reduced Representation Bisulfite Sequencing (RRBS) to analyze differential DNA methylation in the diseased and healthy mucosal tissues of 2 different groups of CD patients: non-surgical and surgical, categorized based on the severity of disease and standard of care needed. Patients in both groups have unique DNA methylation signature compared to the healthy tissue. After removing single nucleotide polymorphisms (SNPs), 1,671 differentially methylated loci were found in the non-surgical and 3,334 in the surgical group of which only 206 were found overlapping in both groups. Furthermore, differential DNA methylation was noted in some of the GWAS associated genes implicated in CD. Also, functional enrichment analysis showed high representation of several key pathways where differential methylations were observed, and these can be implicated in CD pathogenesis. We identified specific DNA methylation patterns in the mucosal DNA of surgical and non-surgical CD patients which indicates evolution of the methylome as the disease progresses from initial to the advance stage. These unique patterns can be used as DNA methylation signatures to identify different stages of the disease.

## 1 Introduction

Inflammatory bowel disease (IBD) represents a growing public health concern due to the increasing incidence worldwide. An increase of 85% from 1990 to 2017 in global IBD cases have been reported (Alatab et al., 2020). Crohn’s disease, one of the most prevalent variants of Inflammatory Bowel Disease is considered as a lifetime and relentlessly progressive disease (Lichtenstein et al., 2004; Freeman, 2014). It can occur at any stage of life and target any part of the gastrointestinal tract but most frequently the distal small intestine (ileum) and the proximal colon (Ranasinghe and Hsu, 2022). In North America alone, the incidence of IBD is 1 case of IBD (CD or Ulcerative Colitis) per 200–250 individuals (approx. 50% CD) resulting in a prevalence of over 1.6M cases per year (Loftus Jr, 2016). CD is initially characterized by inflammation and ulceration which then leads to tissue remodeling including the complications of stricture, fistulization, perforation, and potentially cancer (Rieder and Fiocchi, 2013). As a result, surgical removal of the diseased tissue is needed in 16.3% of the patients at 1 year, 33.3% at 5 years, and 46.6% 10 years after initial diagnosis of the CD to treat severe complications (Frolkis et al., 2013). However, even after removing all visibly active diseased tissue, the incidence of recurrence is still high (Gklavas, 2017). For example, within 1-year after initial surgical procedure clinical recurrence is 20%–37% and endoscopic recurrence is 70%–90% whereas the reoperation incidences are 24% in these patients within 5 years (Penner and Tandon, 2021).

Although the exact mechanism of disease occurrence is not fully understood, previous studies suggest that the disease occurs in genetically predisposed individuals when there is a complex interaction between the altered immune system and dysbiotic microbiome triggered by external environmental factors (Kellermayer, 2012). Genome wide analysis has identified around 140 different genetic loci linked to the inherited nature of Crohn’s disease to make the individuals susceptible to the disease (Park and Jeen, 2019). The genetic variants can result in impairment of the innate immune system which regulates intestinal barrier function to bacteria and other enteral antigens (Cohen et al., 2019). However, these gene variants explain only a very small portion (13.6%) of the CD incidence in patients which (Jostins et al., 2012) leads to the significance of non-inherited factors in the CD occurrence (Adams et al., 2014) and several such factors have already been reported to play a role in disease development, for example, diet, smoking, antibiotics, sanitation and the host microbiome (Hansen et al., 2012).

Epigenetic alteration has been suggested as a possible mechanism to bridge these environmental factors and the related genes variants that could lead to a diseased state (Feinberg and Fallin, 2015). Epigenome analysis has already provided insights on the association between the environment and the predisposed genome in several complex diseases, for example, multiple sclerosis, obesity, and type 2 diabetes mellitus (Ventham et al., 2016). Treatment of CD has been directed to increasing the effectiveness of the intestinal mucosal barrier and decreasing the overactive acquired T cell immune response (Randall et al., 2015). However, understanding the epigenetic changes could lead to development of newer and more effective treatment of CD which could potentially alter the natural history of the disease (Ray and Longworth, 2019). DNA methylation depicts the attachment of a methyl (CH_3_) group at 5′-carbon of pyrimidine ring of the cytosine nucleotide at specific sites (cytosine—guanine CpG) by chemical modification. DNA methyl transferase (DNMTs) enzymes methylate these CpG sites, and this process can be reversed by Ten eleven translocation (TETs) enzymes (Moore et al., 2013). DNA methylation changes are more critical in the promotor region of a gene, either to enhance or downregulate the transcription of that gene (Jones and Takai, 2001). Epigenome wide association studies based on this regulatory mechanism determines the differential DNA methylation at thousands of CpG sites across the genome to find patterns of hypo and hyper methylation in the regulatory region of the genes (Callaway, 2014).

Epigenetic variations due to CD have been reported in several studies. These studies were conducted either by analyzing the blood or diseased intestinal tissues from CD patients. Furthermore, some studies have examined the epigenome of the whole tissue specimen from patients whereas others focused on specific type of cells in these samples (Hornschuh et al., 2021). For example, in the blood methylome studies, sample types included are whole blood, separated B cells, monocytes, T cells and different peripheral blood cells (Nimmo et al., 2012; Adams et al., 2014; McDermott et al., 2016; Ventham et al., 2016; Moret-Tatay et al., 2019; Somineni et al., 2019; Li Yim et al., 2020; Gasparetto et al., 2021). In case of intestinal tissue, sample type used were whole biopsies, fibroblast cells, intestinal epithelial cells, and human adipose stem cells (Harris et al., 2016; Howell et al., 2018; Serena et al., 2020). Most of the epigenome wide studies have identified several differential methylation patterns in the genes linked to CD susceptible genetic loci. DNA methylome signature profiling studies are limited compared to studies with blood and its different cell types. The current study is based on epigenome profiling of the diseased tissues to assess unknown DNA methylation patterns during disease development.

We will utilize RRBS analysis using illumina Novaseq 6000 technology to evaluate the methylome variation in the CD affected mucosal tissue and nearby healthy biopsies from the same patient. This approach will lower the location-based methylome variation across the gastrointestinal (GI) tract and comparison between normal and diseased tissue will be made within the same patient. Furthermore, we divided these patients into two groups based on their standard healthcare plan. Newly diagnosed patients (with mild symptoms of CD) with no surgical procedure required were grouped to non-surgical group and the advanced stage patients where surgery is the only option of cure to remove the diseased tissue were grouped into surgical group.

## 2 Methods

### 2.1 Patients tissue sample collection

The study was conducted under the Institutional Review Board (IRB# 19DHI 2003) and was a collaboration between the Carle Foundation Hospital Urbana IL and University of Illinois at Urbana Champaign. Patients were consented and enrolled at the Digestive Health Institute of Carle Hospital with age in the range between 21 and 34 years and scheduled for their colonoscopies per standard of care for CD evaluation. Patients were divided into 2 groups: Surgical and Non-surgical, diseased and the adjacent healthy tissues/biopsies were collected from each patient during their colonoscopy (non-surgical patients) and surgical procedure (surgical patients). Tissue samples were kept on dry ice at the procedure site and transported to the lab and stored at −80°C till further processing.

### 2.2 Reduced representation bisulfite sequencing

#### 2.2.1 Genomic DNA extraction

Diseased and healthy mucosal tissues collected from the CD patients were used for RRBS analysis. Genomic DNA was extracted and purified from these samples with the Purelink genomic DNA mini kit (Thermofisher, Waltham, MA, United States) per manufacturer’s specification. RNase A treatment was done to eliminate RNA as suggested by the manufacturer and a requirement for RRBS analysis. The concentrations of extracted DNA were measured by the Qubit and the extracted DNA was assessed for its quality by the Fragment analyzer DNA electrophoresis gel.

#### 2.2.2 Library construction

RRBS library construction and sequencing were performed at the Roy J. Carver Biotechnology Center at the University of Illinois at Urbana Champaign using Illumina Novaseq 6000. Libraries were constructed with the Ovation RRBS Methyl-Seq kit from Tecan, CA. Briefly, 100 ng of high molecular weight DNA was digested with MspI enzyme, ligated to sequencing adaptors, treated with bisulfite, and amplified by Polymerase Chain reaction (PCR). The final library concentrations were quantified with Qubit (ThermoFisher, MA) and the average size was determined with a Fragment Analyzer (Agilent, CA). The libraries were then diluted to 10 nM and further quantitated by qPCR on a CFX Connect Real-Time qPCR system (Biorad, Hercules, CA) for accurate pooling of barcoded libraries and maximization of number of clusters in the flowcell.

#### 2.2.3 Sequencing of libraries in the Novaseq 6000

The pooled barcoded shotgun libraries were then loaded on a NovaSeq 6000 SP lane for cluster formation and sequencing. They were sequenced for 100 nucleotides from one side of the DNA fragments. The typical output per lane in the NovaSeq is 400 million reads (SP flowcell) and we obtained approximately 30 million reads per samples. The FASTQ read files were generated and demultiplexed and adapters were trimmed with the bcl2fastq v2.20 Conversion Software (Illumina, San Diego, CA).

### 2.3 Data analysis

#### 2.3.1 Alignment and extraction of DNA methylation calls

After Reduced Representation Bisulfite Sequencing (RRBS) was utilized on the non-surgical group (healthy: n = 3; diseased: n = 3) and the surgical group (healthy: n = 2; diseased: n = 2), Nf-core methylseq pipeline (Ewels et al., 2020) (10.5281/zenodo.2555454) was used for sequence alignment and the extraction of methylation calls (Nextflow version: 22.09.7. edge). The fasta file of human reference genome GRCh38 was retrieved from Gencode (Frankish et al., 2019) (https://www.gencodegenes.org/human/release_42.html) and used to generate the reference genome index. After generating the reference genome index and merging the FastQ files, the diversity adapters were trimmed by the following NuGen protocol (https://github.com/nugentechnologies/NuMetRRBS). Trimmed reads were then aligned to the human reference genome GRCh38 by the default aligner Bowtie2. The first four base pairs were ignored in the alignment due to adapter contamination. The deduplication step was skipped for this analysis. After checking the alignment quality by FastQC (Andrews, 2010), DNA methylation calling was performed by Bismark v0.22.4 (Krueger and Andrews, 2011).

##### 2.3.1.1 Filtering of known SNPs

Before sequencing, bisulfite treatment was used to convert unmethylated cytosine (C) to thymine (T) through uracil (U), while methylated cytosine remained unchanged. Bismark identified the C to T conversion after sequencing alignment and calculated the ratio between the number of Cytosine and the total number of Cytosine and Thymine. Known C-T SNPs were identified as an error source in RRBS data analysis. Here, we used both individual sorted BAM files and merged sorted BAM files generated after alignment to identify the known SNPs by BS-SNPer (Gao et al., 2015). The default settings of BS-SNPer were used (10x coverage was used to align with the parameters in downstream analysis).

##### 2.3.1.2 Exploration of DNA methylation data and comparative analysis

The extracted methylation calls were used as inputs for methyKit (1.22.0) (Akalin et al., 2012) in R (4.2.2) (R Core Team, 2013). Reads with minimum 2x coverage were imported using methylKit:methRead. First, samples were filtered by discarding coverage lower than ten reads and bases with coverage exceeding the 99.9 percentile. Then we normalized the sample data using the default " median " method. C-T SNPs were then removed as further filtering step. After filtering and normalization, the CpGs covered in all libraries were retained for exploratory analysis and examination of global methylation distribution.

Descriptive statistics such as the percentage of CpG methylation and CpG coverage information were explored using methylKit:getMethylationStats and methylKit:getCoverageStats. All reads from ten libraries were merged using methylKit:unit function so that further comparative analysis could be performed. The correlation between the samples was examined using methylKit:getCorrelation function. Clustering and PCA analysis were also performed by methylKit:clusterSamples and methylKit:PCASamples initially. Then customized PCA plots were generated separately for non-surgical and surgical groups to examine the sample separation. Statistical analysis was also used to test the null hypothesis such that no significant difference exists between the non-surgical and surgical groups (25% methylation difference was used as the threshold). Because both non-surgical and surgical datasets are not normally distributed, the Wilcoxon Rank Sum Test was used to compare datasets.

SeqMonk software (Version 1.48.1, https://www.bioinformatics.babraham.ac.uk/projects/seqmonk/) was used to visualize all CpG sites. The genome was divided into consecutive 25-kb probes with a step size of 25 kb. Probes contain at least 100 CpG sites, and each CpG site was covered by at least 2 reads which were retained for plotting the correlation between healthy and diseased mucosal tissue in both groups. We also plotted the within group variation in surgical group.

##### 2.3.1.3 Identification of differentially methylated bases and regions

Before identifying the differentially methylated CpGs, we reorganized methylation data into two groups: Surgical and Non-surgical. The difference in methylation were identified within surgical and non-surgical patient samples. Differential DNA methylation was calculated by comparing the proportion of methylated Cs in our diseased samples *versus* healthy samples in non-surgical and surgical groups. The logistic regression test was used to accomplish the differential DNA methylation calculation: $\log\left(\frac{\pi_{i}}{1-\pi_{i}}\right)=\beta_{0}+\beta_{1}{\text{Treatment}}_{i}$ (Akalin et al., 2012) The resulting *p*-values were generated automatically by multiple testing using the Benjamini–Hochberg FDR method. Differentially methylated CpGs (DMC) were defined as CpG sites that are not overlapping with C-T SNPs with percentage methylation difference >50 and q value <0.01. The type was also specified by “hyper” or “hypo” when extracting the DMCs. Differentially methylated regions (DMRs) are defined using a tiling window of 1 kb resolution comprising of the above CpGs with at least 50% difference in methylation. The global distribution of DMCs across the major chromosomes was generated by ggplot2_3.4.0 (Villanueva and Chen, 2019). The count of DMCs was normalized by the total number of CpGs in each chromosome. The genomic feature track was obtained from Gencode and generated by genomation_1.28.0. The locations of DMCs were summarized in pie charts.

#### 2.3.2 KEGG and GO enrichment analysis

Gene ontology (GO) and Kyoto Encyclopedia of Genes and Genomes (KEGG) are the most widely used methods for functional analysis. Over Representation Analysis was employed to identify whether certain biological processes were enriched in genes based on DMRs. To map genes with DMRs to either KEGG pathways or gene ontology for gene function categorization, ClusterProfiler (Yu et al., 2012; Wu et al., 2021) was utilized. Both the *p*-value cut-off and q value cut-off were set to 0.1 in this analysis. And only the top ten categories were shown in the figures. We also performed pathway enrichment analysis using Reactome (Gillespie et al., 2022), applying a significance threshold set at a *p*-value of 0.05.

## 3 Result

### 3.1 Demographic characteristic and clinical profile of the patients

As shown in Table 1, five CD patients were recruited to yield a mean age of 25.2 ± 5.4 years. CD was diagnosed using standard clinical and endoscopic criteria and characterized based on the Montreal classification and Simple Endoscopic Score (SES-CD). Tissues collected were from diseased ileum paired with adjacent healthy biopsy except one patient where the sampling region was ileocecal valve. These patients were on treatment with different drugs either immunomodulator or corticosteroid or combination of both. In the surgical group (n = 2), both patients had similar ages and biopsies’ locations. While the administered biologics in these patients had different mechanisms of action, their purpose was to modulate the hyperactive immune system. We recognized the sample size is limited; however, our primary focus is on understanding the DNA methylation patterns in surgical patients in severe stage of Crohn’s disease (CD). We anticipate that this study will serve as a foundation for the recruitment of additional patients to enhance the statistical power and the generalizability of the findings in the future.

**TABLE 1:** Crohn’s disease patients clinical/demographic table, non-surgical (NS) and surgical (S) groups.

Patients	Age at enrollment	Gender	Disease classification (Montreal) and simple endoscopic score SES-CD at enrollment	Biopsy’s location		Treatment at enrollment
				Diseased	Healthy	
NS-01	22	male	SES-CD 10	ileum	ileum	Imuran 75 mg daily; entocort EC 9 mg daily; stelara 390 mg every 8 weeks
NS-02	34	male	SES-CD not noted; baseline scope—“The colon (entire examined portion) appeared normal. The ileocecal valve severely ulcerated and strictured unable to traverse into the terminal ileum.”	ileocecal	ileocecal	none
NS-03	21	male	SES-CD 4, Montreal Classification A1L1p	ileum	ileum	imuran 150 mg daily
S-01	27	female	Montreal A2 (L3 + L4) B3p (estimate using clinical summaries)	ileum	ileum	Stelara 260 mg once prior to enrollment
S-02	22	male	SES-CD 11	ileum	ileum	Humira 40 mg every other week; budesonide 9 mg daily

### 3.2 Quality control on sequence alignment

Reduced Representation Bisulfite Sequencing (RRBS) produced a total of 2.74 × 10^8^ paired end reads for ten libraries. After adapter trimming, 2.59 × 10^8^ reads remained (Supplementary Table S1). By employing the default Bowtie2 aligner, we achieved an average unique alignment rate of 73.51% to the human reference genome GRCh38. Additionally, we conducted a comparison of mapping efficiency between two aligners: Bowtie2 and Hisat2. The average mapping efficiency of Hisat2 was slightly lower at 72.97% when compared to Bowtie2’s 73.51%. Consequently, we opted to proceed with Bowtie2 for the subsequent downstream analysis. (Supplementary Table S2) An average of 65.63% duplicate reads were detected within the sequences, which is typical for RRBS data (Supplementary Table S2).

### 3.3 Identification of known SNPs

C-T SNPs were identified in RRBS data. 65,885 unique C-T SNPs were found across individual or merged BAM files. These were removed when visualizing the global methylation patterns and performing downstream analysis because they do not represent the actual C/T substitutions by bisulfite conversion.

### 3.4 Exploratory statistics and global DNA methylation patterns

**FIGURE 1:**
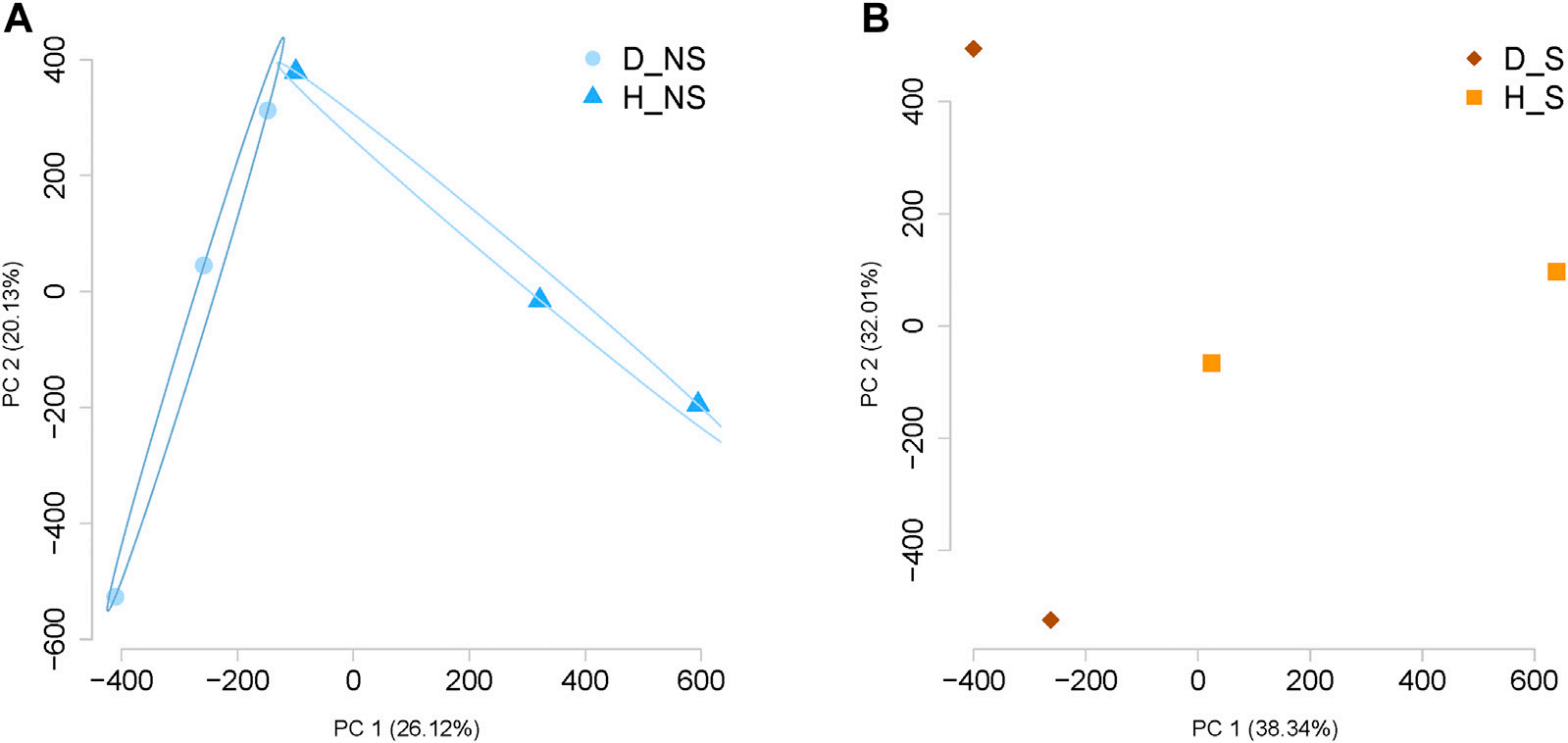
Principal Component Analysis (PCA) for the data per group. **(A)** PCA demonstrates the separation of the data between diseased non-surgical patient samples and healthy non-surgical diseased patient samples (n = 3). **(B)** Separation between the samples was visualized by PCA analysis between diseased and healthy surgical patient samples (n = 2). NS: Non-surgical patient; S: surgical patient; D, patient with Crohn’s disease; H, healthy patient sample.

Around 2.6 × 10^6^ CpG sites were identified within each sample (Supplementary Table S2). The histograms for percentage CpG distribution showed that most of the loci had either high methylation (>85%) or low methylation percentage (<5%) within each sample. The read coverages of CpG per base within each sample were less than 70 (Supplementary Figure S1). High pair-wise Pearson’s correlation coefficients were detected consistently between the groups (Supplementary Figure S2). Principle Component Analysis shows a clear separation between healthy and diseased patient samples per group (Figure 1). Further, combining all samples together on a PCA plot showed separation between healthy and diseased (Supplementary Figure S3). Statistical analysis was also used to confirm a significant difference between non-surgical and surgical groups. This analysis involved combining the methylation data from both groups to compare their respective methylation patterns. We visualized our global methylation patterns in SeqMonk. The scatter plots are shown in Supplementary Figure S4.

**FIGURE 2:**
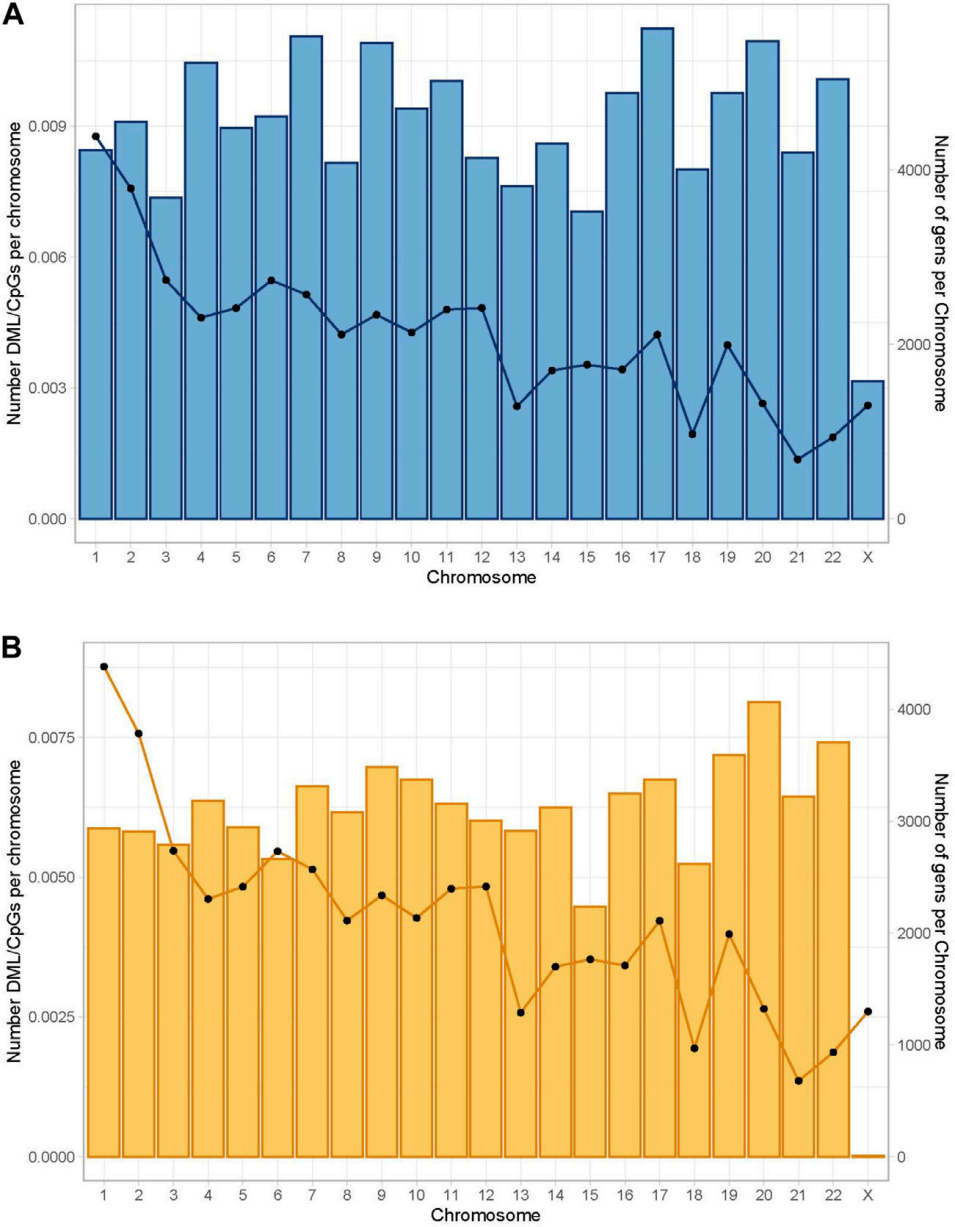
Global distribution of differentially methylated CpGs per chromosome. The bars represent the number of differentially methylated CpG loci normalized by the number of CpG sites per chromosome. The lines represent the number of genes per chromosome. Differential percentage methylation cut off at 25. **(A)** Global distribution in the non-surgical group. **(B)** Global distribution in the surgical group.

A total of 1,109,247 CpGs were recognized in surgical and non-surgical 10x coverage combined data after removing C- T SNPs. This represents 3.4% of the total CpG loci in the human genome GRCh38 (Human genome GRCh38 contains around 32.28 million CpGs) (Gershman et al., 2022). Only 1,030 bases (0.0928% of total identified CpGs) were removed due to the false characterization (C-T mutations). Then we explored the percentage of differentially methylated CpGs (DMC) per chromosome. DMC were present in all major chromosomes, and we observed the highest normalized DML ratios in chromosome 1 in both groups (Figure 2).

**FIGURE 3:**
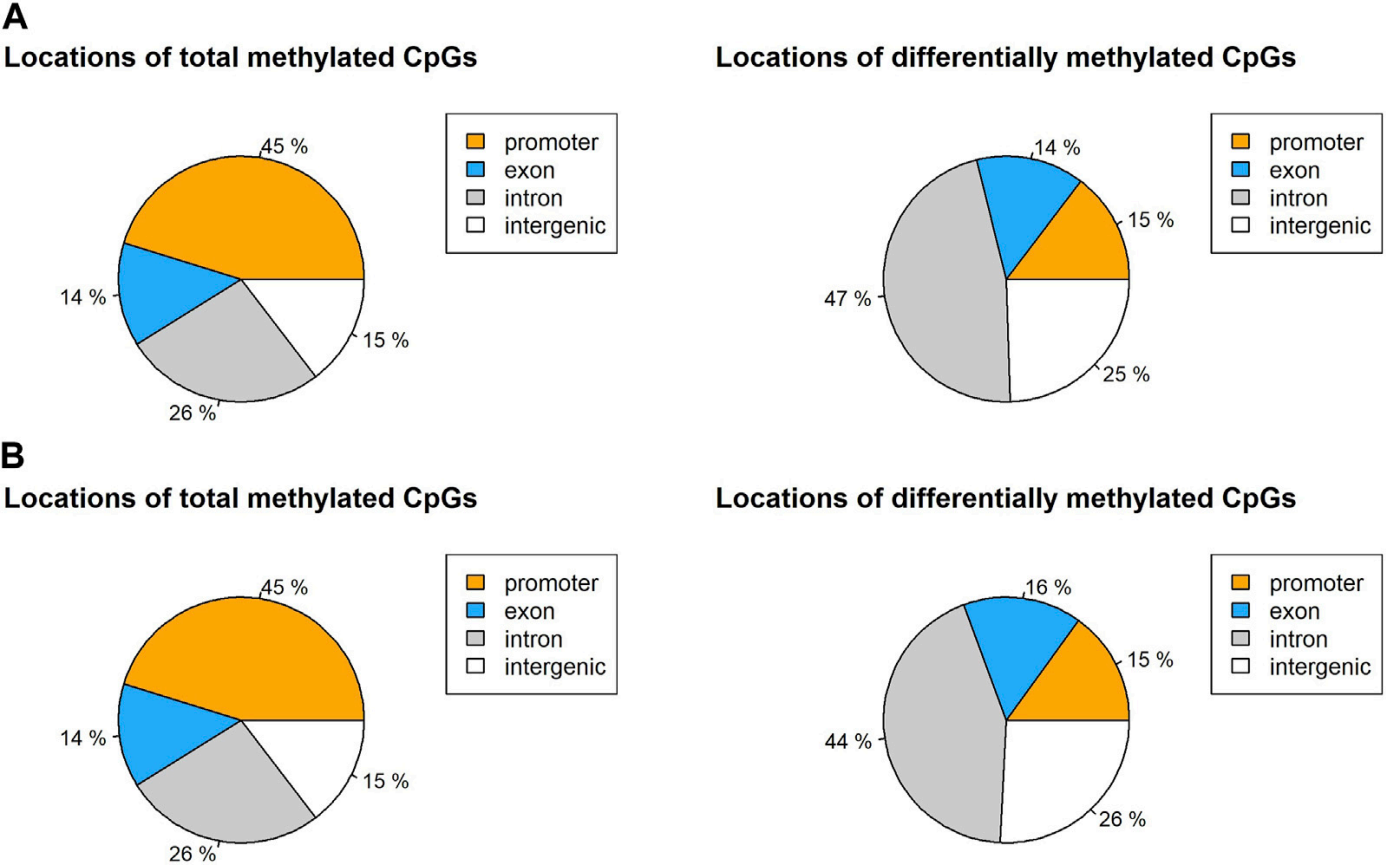
Locations of total CpG loci with 10x coverage and differentially methylated CpGs in **(A)** non-surgical group and **(B)** surgical group.

Differential methylation at each base was calculated by a logistic regression-based method. Then it was filtered by q-value at a significance level of 0.01 and a percentage methylation difference greater than 50%. In the non-surgical group, most CpGs (1,107,560) were either lowly or mildly methylated (methylation percentage difference smaller than 50%). Only 1.52% of the CpGs (1,687) are highly methylated (methylation percentage difference equal to or larger than 50) (Supplementary Figure S5). We observed 3.07% of the CpGs (3,408) to be highly methylated in the surgical group. Among all identified CpG loci in non-surgical and surgical groups, 45% of the CpGs were located in the promoter region while 26% of the CpGs were within an intron (Figure 3, Supplementary Table S3).

### 3.5 Identification of differentially methylated bases and differentially methylated regions

**FIGURE 4:**
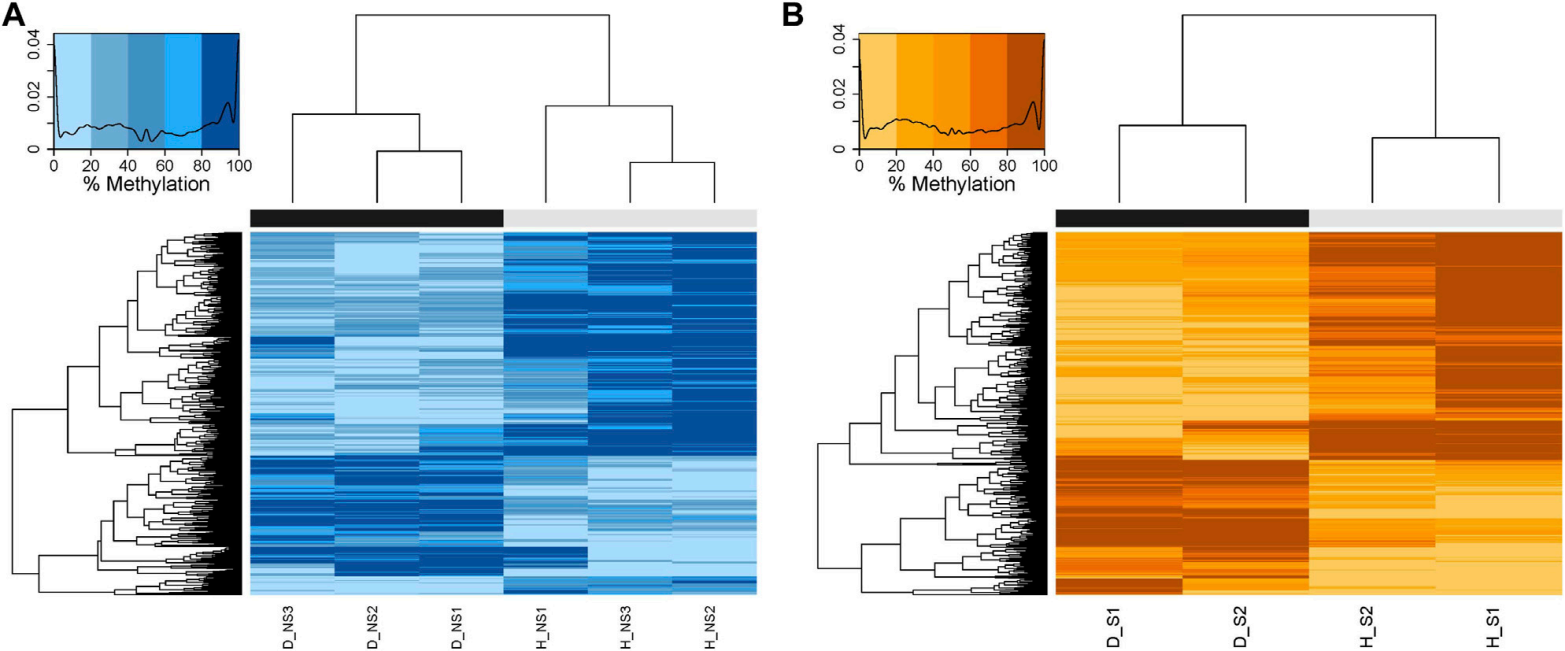
Heatmaps showing the hierarchical clustering of DMCs with similar percentage DNA methylation levels created by calculating the Euclidean distance between the differentially methylated bases. The darker the color, the higher the percentage of methylation. The black bar represents the diseased patient samples, and the grey bar represents the healthy patient samples. **(A)** NS: Non-surgical patient **(B)** S: surgical patient.

1,671 and 3,334 CpG sites were initially identified as differential methylation bases in non-surgical and surgical groups, respectively, using a 50% differential methylation percentage cut-off. After removing the seven overlapping C-T SNPs in the non-surgical group and the ten overlapping C-T SNPs in the surgical group, the remaining CpG loci were used for downstream analysis (1,664 and 3,324, respectively). Within the differentially methylated CpG sites (DMCs) in the non-surgical group, there are 552 hypermethylated and 1,112 hypomethylated sites. The surgical group consists of 1,235 hypermethylated and 2,089 hypomethylated sites (Figure 4). We also identified differentially methylated regions (DMRs) by using the above CpGs. There were 50 DMRs in the non-surgical group and 106 DMRs in surgical group (Supplementary Figures S6A, B and Supplementary Tables S4, S5). We did not observe significant methylation difference between the healthy tissues in two groups and diseased tissues between two groups (Supplementary Figures S6C, D).

### 3.6 Similarity and difference between non-surgical and surgical groups

**FIGURE 5:**
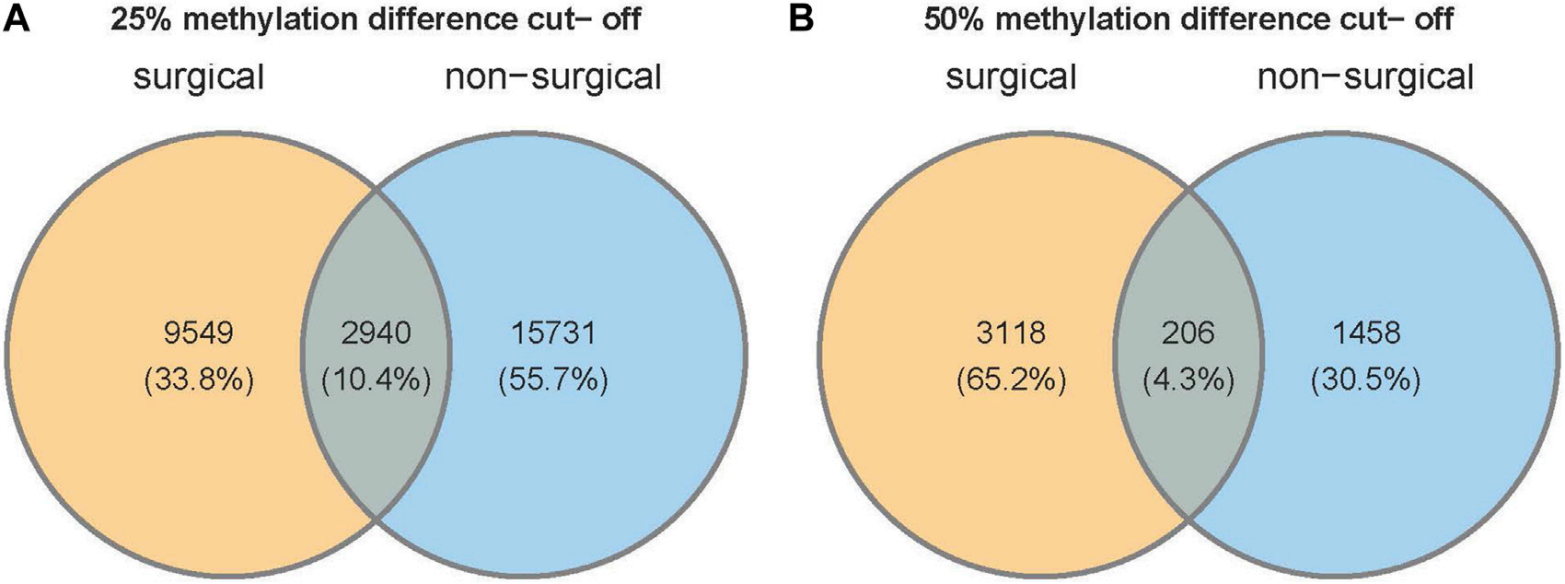
Venn diagram shows the number of unique CpG sites and overlapping CpGs with two different thresholds of **(A)** 25% or **(B)** 50% methylation difference.

We identified a total of 1,109,247 of CpGs in both non-surgical and surgical groups. 18,706 CpGs were somewhat methylated (25% methylation difference cut-off) in non-surgical patient samples, which is higher than that in surgical patient samples (12,489). Only 2,940 CpGs (10.4%) were present in both groups (Figure 5). If we change the threshold to a 50% methylation difference, 1,671 DMCs were found in the non-surgical group and 3,334 in the surgical group. Only 206 DMCs (4.3%) were found overlapping in both groups (Figure 5). The amount of differentially methylated bases (3,334) in the surgical group is more than double that in the non-surgical group (1,671).

### 3.7 Crohn’s disease-linked genes and confirmation of alteration of methylation in our groups

After identifying the DMCs, we were interested in investigating methylation levels in CD-linked genes shown by previous GWAS studies. We selected a few of these genes (Supplementary Table S6) and analyzed the methylation pattern in the healthy and diseased tissue samples summarized in Table 2.

**TABLE 2:** DNA Methylation state of Crohn’s disease-related genes. NS, Non-surgical patients; S, surgical patients.

Gene symbol	Group	Location	Chr	Start/End	Methylation difference (%)	Methylation state	Gene expression in CD
IRGM	NS	promoter exon	chr5	150,846,720	29.52775476	Hyper	Up or downregulated variant dependent (Ajayi et al., 2019)
			chr5	150,846,746	29.991715	Hyper	
			chr5	150,846,762	40.56338028	Hyper	
	S	promoter exon	chr5	150,846,886	39.91304348	Hyper	
CARD9	NS	promoter	chr9	136,374,386	−36.65546846	Hypo	Variant upregulated (Luo et al., 2020)
		intron	chr9	136,380,070	30.4981452	Hyper	
			chr9	136,380,071	43.28063241	Hyper	
MUC2	NS	intron	chr11	1,082,168	−33.29787234	Hypo	Up (Cari et al., 2023)
		exon	chr11	1,083,816	−30.85048754	Hypo	
	S	intron	chr11	1,077,402	−44.58364038	Hypo	
			chr11	1,081,571	−41.94444444	Hypo	
			chr11	1,081,601	−45	Hypo	
NOD2	NS	intron	chr16	50,719,712	−36.54051173	Hypo	
	S	exon	chr16	50,715,637	37.36805067	Hyper	
		intron	chr16	50,719,712	−63.60779384	Hypo	
			chr16	50,719,717	−44.68887492	Hypo	
TYK2	NS	intron	chr19	10,353,462	29.5194508	Hyper	
	S	intergenic	chr19	1,0,381,760	34.47368421	Hyper	
			chr19	10,388,056	−61.29032258	Hypo	
DMRs mapped to CD linked genes							
MUC4	NS	promoter exon intron	chr3	195,810,001/195,811,000	−61.7433	Hypo	Up (Dorofeyev et al., 2013; Li et al., 2023)
		exon intron	chr3	195,809,001/195,810,000	−54.7963	Hypo	
	S	exon intron	chr3	195,809,001/195,810,000	−51.6707	Hypo	
IL10RB	S	intron	chr21	33,290,001/33,291,000	−52.5686	Hypo	
SLC15A1	S	intergenic	chr13	98,776,001/98,777,000	−51.5779	Hypo	Up (Ingersoll et al., 2012)
PIK3R2	NS	exon intron	chr19	18,155,001/18,156,000	−58.7821	Hypo	
	S	exon intron	chr19	18,155,001/18,156,000	−51.2262	Hypo	

Nucleotide-binding oligomerization domain-containing protein 2 (NOD2), the first confirmed gene in CD which is involved in sensing microbial components, regulating inflammatory factors, and apoptosis (Eckmann and Karin, 2005) was hypomethylated in the intron region of the gene in both non-surgical and surgical groups, and hypomethylation level was twice in surgical group, whereas hypermethylation was observed at one CpG site in the exon region in the non-surgical samples. Furthermore, we observed mild hyper-methylation on the Immunity Related GTPase M (IRGM) gene’s promoter or exon region in both non-surgical and surgical groups. On the contrary, only mild hypo-methylation was detected in mucin 2 (MUC2) gene.

The change in methylation level of CpGs on the Caspase recruitment domain-containing protein 9 (CARD9) gene was only detected in the non-surgical group. The CpG site in the promoter region of this gene had a mild hypo-methylation, while the other two CpG sites in the intron region were mildly hypermethylated. We observed methylation changes in one of the CpG sites on the intron of this gene in the non-surgical group and two CpG sites in the intergenic region of the surgical group. Significant hypo-methylation was detected in one of the surgical group’s CpG site (Table 2).

Further, we observed methylation change in one of the CpG sites on the intron of the non-receptor tyrosine-protein kinase (TYK2) gene in the non-surgical group and two CpG sites in the intergenic region of the surgical group. Significant hypo-methylation was noted in one of the surgical group’s CpGs (chr19 10388056) (Table 2).

Based on the DMRs, we have identified several genes which are linked to IBD to be differentially methylated in diseased tissue compared to the healthy biopsies. In surgical patients hypomethylation pattern was observed in the following genes: Mucin 4 (MUC4) at exon/intron, Interleukin 10 Receptor subunit beta (IL10RB) at intron and Solute Carrier Family 15 Oligopeptide Transporter Member 1 (SLC15A1) at intergenic regions. In non-surgical patients MUC4 was hypomethylated in 2 different regions: promotor/exon/intron and exon/intron. Interestingly Phosphoinositide-3-Kinase Regulatory Subunit 2 (PIK3R2) gene which is considered as tumor driver was hypomethylated (exon/intron region) both in surgical and non-surgical patients (Table 2).

### 3.8 Enriched pathways/cellular processes and gene ontologies were identified in our groups

**FIGURE 6:**
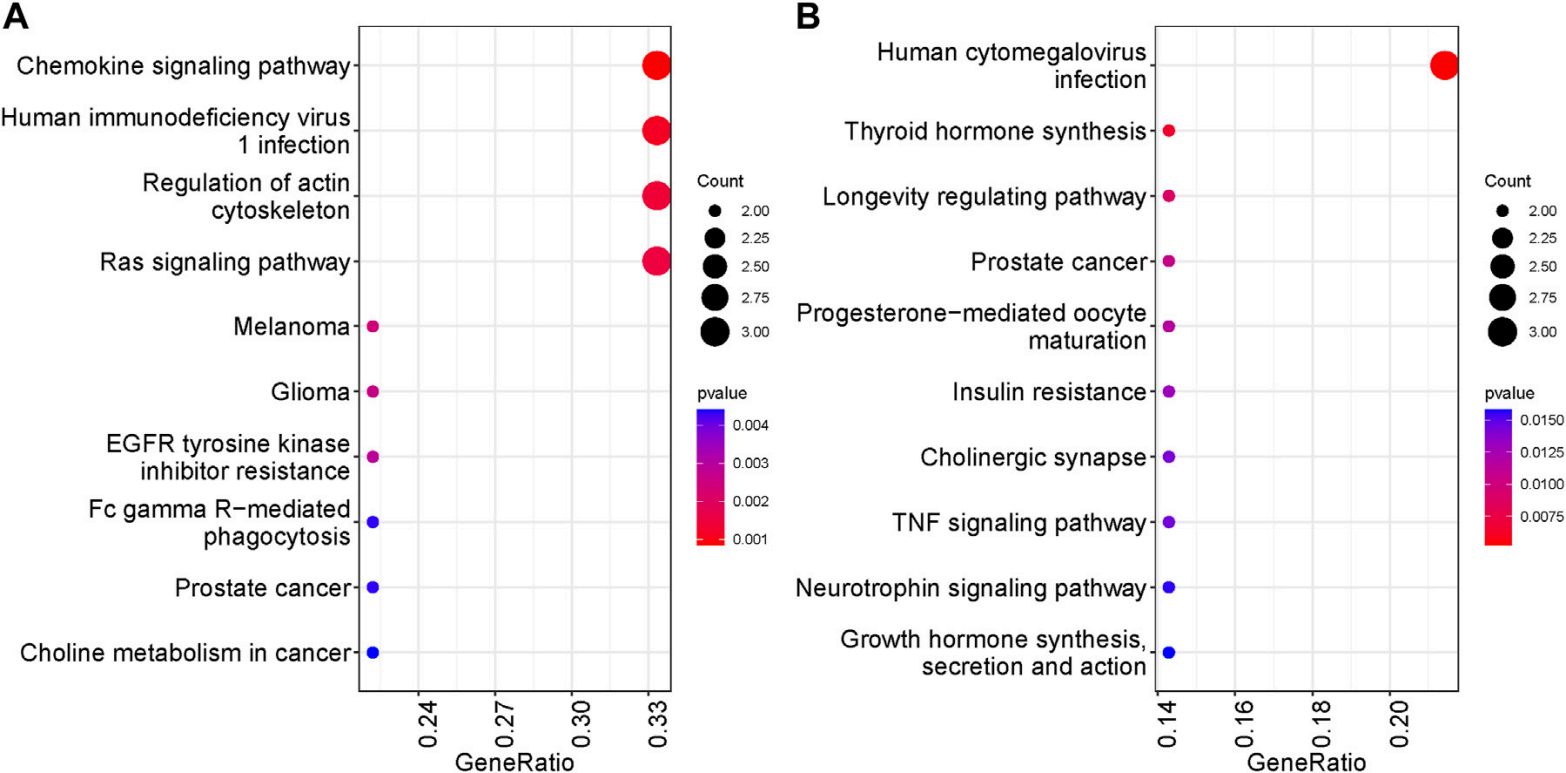
Top ten KEGG pathways represented by genes with DMRs. The significance was based on *p* values. **(A)** KEGG analysis in NS group **(B)** KEGG analysis in S group.

**FIGURE 7:**
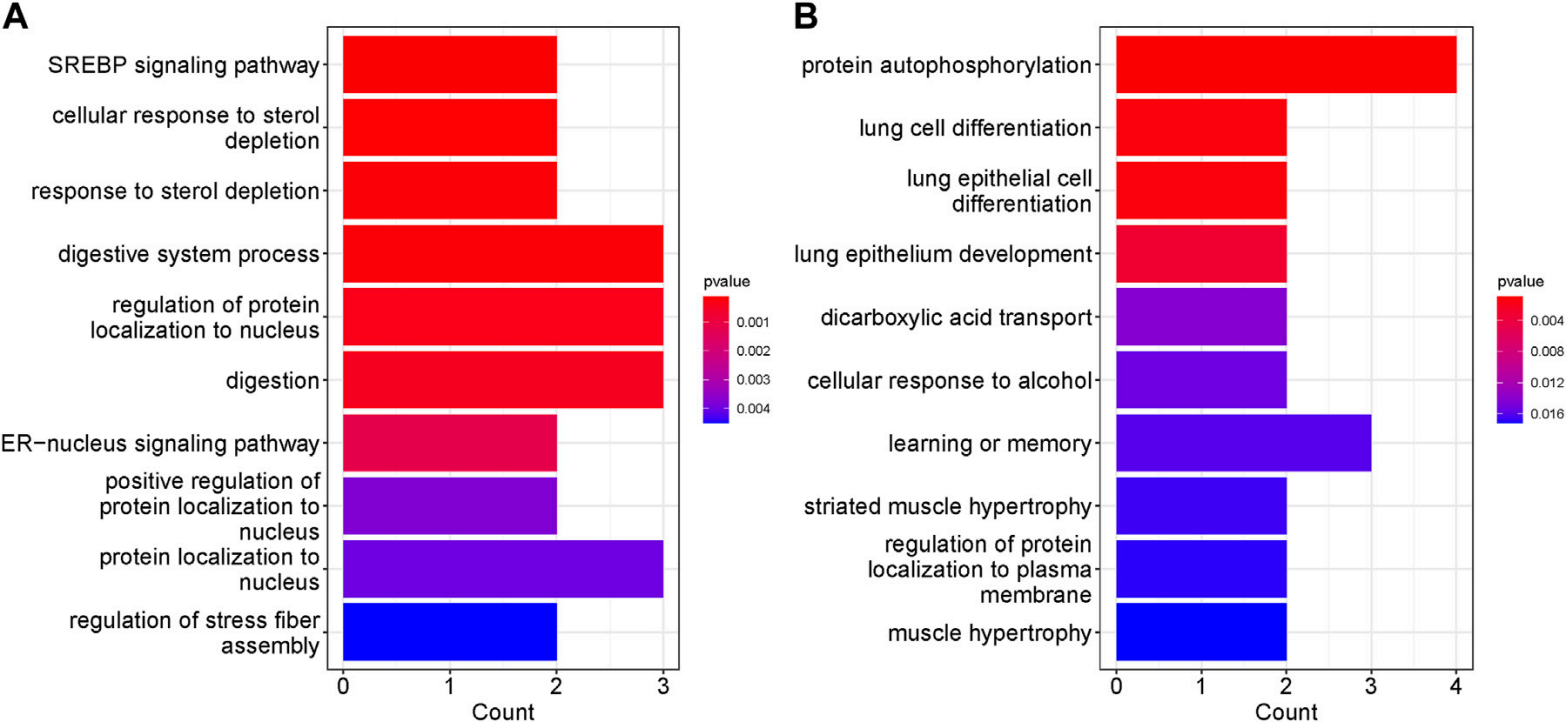
Top ten GO terms represented by genes with DMRs. The significance was based on *p* values. **(A)** GO enrichment in NS group **(B)** GO enrichment in S group.

We performed KEGG and GO enrichment analyses on genes with differentially methylated regions (DMR) to determine whether these genes were associated with specific cellular processes or molecular functions. Chemokine signaling pathway, HIV-1 infection and regulation of actin cytoskeleton have the highest representation in the non-surgical group, while the Human cytomegalovirus infection was the most represented in the surgical group. Pathways related to TNF signaling and several hormones synthesis, resistance and their mode of action have representation in surgical group. In both non surgical and surgical groups, prostate cancer KEGG pathway was found to be common (Figure 6). We also conducted pathway enrichment analysis using Reactome. The overrepresentation analysis revealed that the prominent pathways enriched in the non-surgical group encompass Signal Transduction, Disease, Metabolism of Proteins, and Programmed Cell Death. In contrast, the key pathways overrepresented in the surgical group involve Signal Transduction, Disease, Metabolism of Proteins, DNA Repair, and Gene Expression. Notably, these findings align with our KEGG enrichment results (Supplementary Figure S7). In the non-surgical group, the gene ontology related to digestive system process, protein localization to the nucleus and digestion showed highest representation (Figure 7A) whereas protein autophosphorylation was dominant in surgical group (Figure 7B). However, none of the gene ontologies were shared between the two groups.

## 4 Discussion

We present novel data comprising of the alteration in DNA methylome between diseased and healthy Crohn’s disease patient tissue samples in both non-surgical and surgical groups. We separately identified 1,664 and 3,324 differentially methylated sites in the non-surgical and surgical groups and have shown changes in DNA methylation level in Crohn’s disease mucosal tissues. Furthermore, we found that the number of DMCs in surgical patient samples is almost double than that in non-surgical patients, and only 4.3% of DMCs were present in both groups. We also observed methylation level changes in several closely CD-linked genes in diseased patient samples of both groups. These facts confirm our hypothesis that the alteration in methylation has a high relevance to the development of the disease. Our study establishes a foundation for future research in understanding the underlying molecular mechanisms of the epigenome evolution in CD and provides insight on the methylation programming during different stages of the CD.

### 4.1 Previous studies exploring methylation profiling in CDs and bioinformatics analysis of RRBS

Several previous studies have explored genome-wide methylation profiles in different tissues from CD patients. For example, Li et al. has screened genome-wide DNA methylation in adult CD patients’ penetrating lesion intestinal mucosa tissue and identified 5,200 DMCs, mostly in the gene body (Li et al., 2021) (Table 3). Yim et al. evaluated methylation profiling of peripheral blood mononuclear cells (PBMCs) from active and remissive patients (Li Yim et al., 2020) and concluded that DNA methylation variation is associated with the stage of CD (Table 3). All previous studies confirmed a relationship between CD development and epigenetic variation in different tissues, but none reported the percentage of CpGs methylated and the global methylation pattern. While there has been a fair amount of effort to explore the linkage between the variation in methylation and CD activity, we are the first to use RRBS analysis to assess CpG sites in CD patients and have expounded on global methylation patterns. We also compared the DNA methylome alternation in two groups: CD patients without surgical procedure (initial stage of the disease) and CD patients after surgery (advance stage of the disease).

**TABLE 3:** Studies exploring DNA methylation responses in patients with Crohn’s disease.

Study tissue	Sequencing method	Percent CpGs methylated	Global methylation differences	Differential methylation (hyper/hypo methylated)		Genome features impacted	Gene functions enriched at differentially methylated sites	Connection to transcription
intestinal mucosa tissue **(Li et al., 2021)**	Illumina MethylationEPIC (850K) array	NR (not reported)	NR	5,200 DMPs (2,978/2,222)	The majority of hypermethylated and hypomethylated sites are in the gene bodies		GO analysis showed that differential DNA methylation sites were enriched in the positive regulation of the apoptotic process and the positive regulation of interleukin-8 production in the biological approach. Pathway analysis of differential DNA methylation sites with the KEGG database showed that the differentially expressed sites were mainly concentrated in signal pathways associated with IBD	The differentiated methylated regions directly affected gene expression. (Gene expression dataset downloaded from NCBI GEO datasets
circulating monocytes (CD14 cells) **(Li Yim et al., 2020)**	HM450	NR	NR	15 DMGs (11/4)	Methylation takes place in the promoter region and gene bodies		The STRING database indicates that DMGs do not represent clear functional modules or cellular pathways, but some are implicated in immunological functions	NR
colon specimens **(Kim et al., 2020)**	HM450	NR	NR	3,178 DMPs (2016/1,162)	For hypermethylated regions, 35% were in promoter regions, 35% in the gene body, and 27% in intergenic regions. For hypomethylated areas, 35% in the intergenic region, 32% in the promoter region, and 31% in the gene body		GO analysis reveals several pathways associated with the hypermethylated genes were related to the bleb assembly, regulation of actin filament-based processes, and steroid metabolic processes. Hypomethylated genes are related to leukocyte activation and are involved in immune response, lymphocyte proliferation, and cytokine production	NR
circulating immune cells **(Somineni et al., 2019)**	WGBS	NR	NR	1,189 DMPs (976/213)	DMPs are more likely to occur in gene bodies and CpG shelves and less likely in gene promoters and CpG islands and shores		Pathway enrichment analysis revealed pathways relevant to immune function, including tumor necrosis factor-a, JAK-STAT, Ras-related protein 1, and phosphoinositide 3-kinase–Akt signaling, and inflammation, such as the interleukin 17 signaling pathway, cytokine–cytokine receptor interaction, and chemokine signaling	162 of 585 differentially methylated genes (28%) were differentially expressed. (RNA-Seq). The direction of effects between DNA methylation and gene expression changes in relation to Crohn’s disease appears to be context-dependent, irrespective of the position of the CpG site in the associated gene
peripheral blood mononuclear cells **(Ventham et al., 2016)**	HM450 (Illumina HumanMethylation450K Beadchip), WGBS	NR	NR	412 DMPs in Crohn’s Disease with compared to control; four CD-associated DMRs (VMP1, ITGB2, WDR8 and CDC42BPB)	Of the 4 CD-associated DMRs, VMP1 and WDR8 were in gene body, while ITGB-8 was in the 5′-Untranslated region. Most DMPs are located in the gene body		Of all DMPs, 54 significantly enriched GO terms were found, a large proportion of which relate to immune function	Hypermethylation within the TXK gene between the 5′untranslated region and first exon region was associated with reduced TXK expression in CD8^+^ T cells but no other cell types. Cell specificity plays an important role in methylation and gene expression
purified fibrotic human intestinal fibroblasts **(Sadler et al., 2016)**	MiGS	NR	NR	1982 DMRs (1,180/802)	43.1% of mapped differential DNA methylation occurred within introns, 48.4% occurred within intergenic regions, while only 2.9% occurred within exons, and 2.7% within promoters		Of all the DMRs, the open sea regions (loci greater than 4 kb from CpG islands) contained most of the differential non-CpG island methylation (86.3% and 87.7%)	RNA-seq analysis identified the fibrosis-associated changes in the HIF transcriptome associated with changes in DNA methylation
peripheral blood **(Li Yim et al., 2016)**	HM450	NR	NR	4,287 DMPs (3,338/949)	A statistically significant difference in the DMP distribution for the transcription start sites (TSS1500 and TSS200), the gene body, the first exon, the 3′untranslated region (3′UTR), and the intergenic region were observed		GO enrichment performances showed enriched processes for immune response, leukocyte activation, and neutrophil chemotaxis	NR

### 4.2 CpG methylation pattern and methylation level of CD-associated genes

After removing the C-T SNPs, we examined the global CpG methylation pattern and identified 1,109,247 CpG sites in our 10x coverage data. 1030 C-T SNPs (0.0928%) were removed before further analysis. Even though C-T SNPs are one of the error sources in RRBS analysis (Gao et al., 2015), we did not see a difference in our case. Before removing the SNPs, we identified 1,671 and 3,334 significantly methylated bases in the non-surgical and surgical group, respectively. After removing the SNPs, 1,664 and 3,324 methylated CpGs were retained.

The global CpG methylation patterns are similar in both groups. Almost half of the CpGs are in the promoters, and over a quarter of the CpGs are in the introns. Considering the DMCs, most of the DMCs were found in introns, and over 25% of DMCs are in the intergenic region. Only 15% of the DMCs were found in the promoters (Figure 5). The DMC distribution across the major chromosomes is also similar in both groups. However, among the DMCs, our findings show that only 4.3% of DMCs were present in both groups, indicating that the pattern of methylation changes is quite different between non-surgical and surgical patients.

To assess any significant methylation changes in Crohn’s disease-related genes, we first selected a few genes with established linkage to CD and evaluated their methylation level. The RRBS analysis shows some methylation changes in IRGM, CARD9, MUC2, NOD2, TYK2, MUC4, IL10RB and SLC15A1. Significant hypomethylation was observed on the CpG site in the intron of NOD2 and the CpG site in the intergenic region of TYK2 in the surgical patients. TYK2 is essential in JAK/TYK2 signaling of inflammatory bowel disease (Danese and Peyrin-Biroulet, 2021) NOD2 is the first risk gene that has been identified for CD (Eckmann and Karin, 2005; Yamamoto and Ma, 2009). Our data shows that three locations on the gene body of NOD2 were hyper or hypo methylated. The methylation level of CpG located on chromosome 16, position 50,719,712, increased with the development of CD. IRGM plays a protective role in inflammatory diseases and decreases inflammation in the gut by autophagic degradation of inflammasome (Mehto et al., 2019). Several loci in the IRGM gene have been reported to dysregulate the expression of the gene and the autophagy pathway (Ajayi et al., 2019). Patterns of hypermethylation at different CpG sites were noted in this gene which can possibly lead to its downregulation and its function. CARD9 gene plays a major role in sensing of pathogens in the gut to regulate activation of the innate immune system (Luo et al., 2020). Intron region of the gene was hypermethylated whereas hypomethylation was observed in the promotor region of NS patients. Differential methylation has been reported for this gene in ulcerative colitis at different CpG sites (Cooke et al., 2012). MUC2 gene, which provides a protective barrier between the gut epithelial surface and the lumen (Allen et al., 1998) was mildly hypomethylated in the diseased tissues, in contrast to the non-significant hypermethylation pattern in the pediatric CD patients (Kraiczy et al., 2016). Age could be the possible factor for this variation since we collected tissue specimens from the adults.

Several CD associated genes were observed in DMR regions. For example, MUC4 encoding a transmembrane protein, play its role in maintaining GI epithelium and imbalance expression can contribute to IBD (McGuckin et al., 2011) was hypomethylated both in surgical and non-surgical groups. Hypomethylation patterns in different regions of the MUC4 in the current work support its upregulated expression in IBD studies reported previously (Dorofeyev et al., 2013; Li et al., 2023). An animal model study showed that mice deficient in Muc4 gene were resistant to experimental colitis and colorectal cancer linked to colitis (Das et al., 2016). SLC15A1 gene encoding di/tripeptide transporter (PepT1) protein is primarily expressed in brush border membranes and highly express in colonic region during IBD which contributes to the pathogenesis of inflammation (Ingersoll et al., 2012). It was hypomethylated in surgical patients which can be a reason for its upregulation in the inflammation. PIK3R2 which is not directly involved and reported in CD but has hypomethylated exon/intron regions in both surgical and non-surgical group. This gene is considered as tumor driver and has reported in tens of different cancer (Liu et al., 2022). In colorectal cancer PIK3R2 contribute to the tumor proliferation and metastasis (Cortés et al., 2012). It will be of interest to explore the implication of this oncogene in IBD.

### 4.3 Enriched pathways/cellular processes and gene ontologies

We further conducted functional analysis to identify the pathways and gene ontologies associated with genes exhibiting DMRs. We observed a diverse range of biological processes and molecular functions represented in both groups. In the non-surgical group, the chemokine signaling pathway stood out with highest representation. Chemokines are small proteins which can attract immune cells (leukocytes) to the inflamed sites in IBD affected intestinal lesions (Singh et al., 2016). Also, this pathway is a potential therapeutic target in IBD (Trivedi and Adams, 2018). Additionally, the regulation of Actin Cytoskeleton pathway was prominently featured, as it is involved in various cellular processes such as cell migration, adhesion, shape changes, and intracellular transport (Svitkina, 2018). In surgical patients human CMV infection related pathway has the highest representation. CMV infection is frequently observed in the IBD patients with a poor outcome—compromised immune system in IBD can be a cause to this viral infection (Criscuoli et al., 2006). TNF signaling pathway was also observed which play integral role in IBD and is a target for anti-TNF biologics to lower the inflammation (Bradford et al., 2017).

Some of the represented GO in non-surgical group were, digestive system and digestion processes which are linked to the GI tract health and can lead to the CD symptoms. In surgical patients, muscle hypertrophy and striated muscle hypertrophy were somewhat represented. In CD stricturing, the smooth muscle hypertrophy and hyperplasia are mainly responsible and can be cause for these strictures in the diseased condition (Chen et al., 2017).

### 4.4 Conclusion

To conclude, we investigated the DNA methylation profiles in the mild and highly inflamed mucosal CD tissues. Our findings reveal that surgical patients at advanced stages of Crohn’s disease have different epigenomic pattern suggesting a clear linkage between the development of the disease and alternations in the DNA methylome. Our study also found that most of the differential methylation events occur in the genic regions, which is consistent with previous research. Furthermore, we observed altered methylation levels in several key CD-linked genes, providing additional evidence that epigenetic changes play a crucial role in the underlying molecular mechanisms. Despite the identification of unique methylome signature in surgical/non-surgical CD patients we would like to highlight some limitations in the current work. For example, limited sample size in our case makes it challenging to further conclude on the complex pathways in the disease conditions. Further, the downstream functional analysis such as transcriptomic and proteomic studies of these methylome variations could provide insights on molecular mechanisms and pathways implicated in CD. Further work on the temporal evolution of the methalome could shed light on the evolution of the disease over the disease course.

Overall, our work highlights the importance of considering epigenetic changes in the treatment of Crohn’s disease. By elucidating the complex interactions between genetics, epigenetics, and disease development, our work could lead to development of therapeutic strategies that could alter the course of Crohn’s disease.

## Data Availability

Publicly available datasets were analyzed in this study. This data can be found here: https://www.ncbi.nlm.nih.gov/bioproject/994045.
