# Supplementary material for: Mucosal DNA methylome alteration in Crohn’s disease: surgical and non-surgical groups
Source: Front Genet. 2023 Nov 17;14:1244513. doi: 10.3389/fgene.2023.1244513 (PMC10691104; doi:10.3389/fgene.2023.1244513)
Supplement: Supplementary file 2 [file Image1.pdf]

Supplementary Material

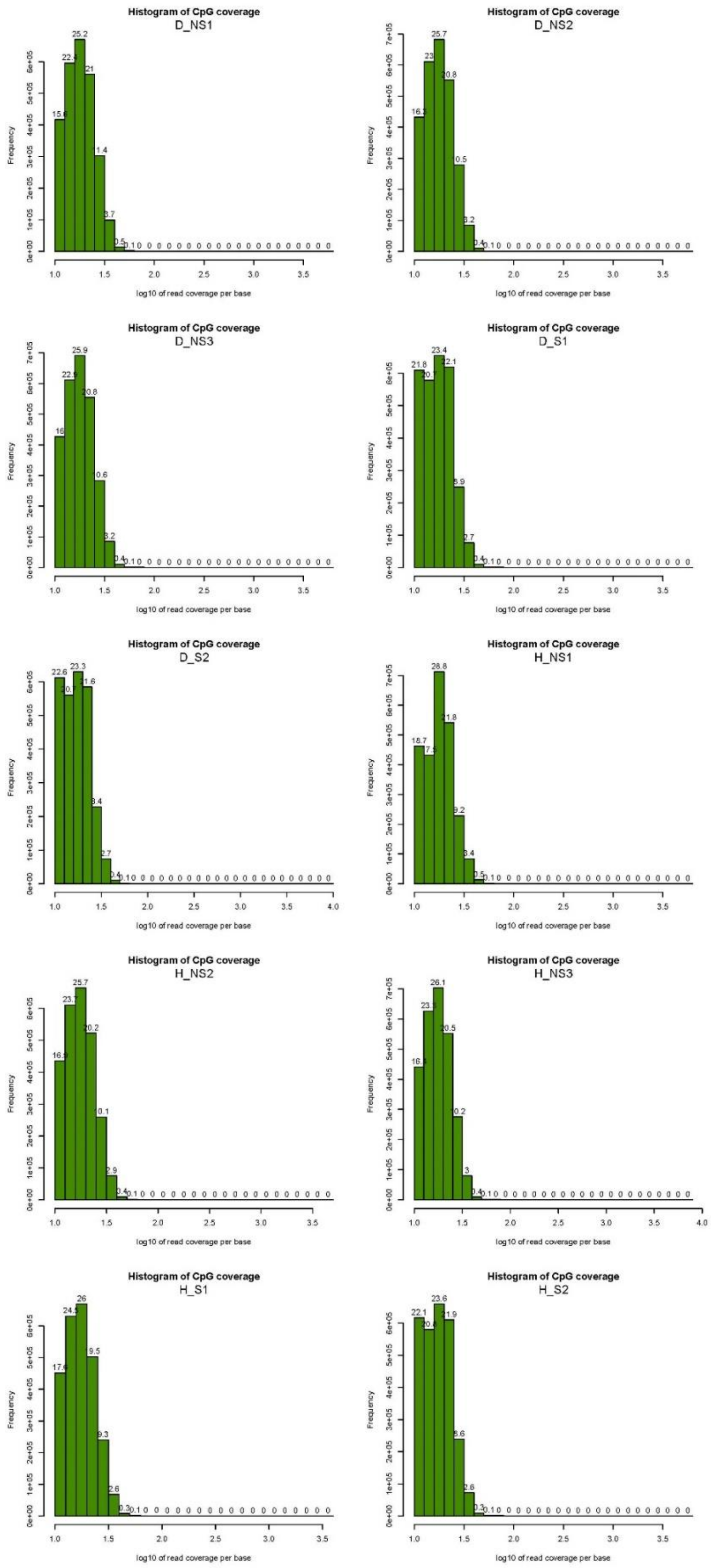

Figure S1. Descriptive Statistics per sample, histogram of CpG coverage of diseased non-surgical patient sample.

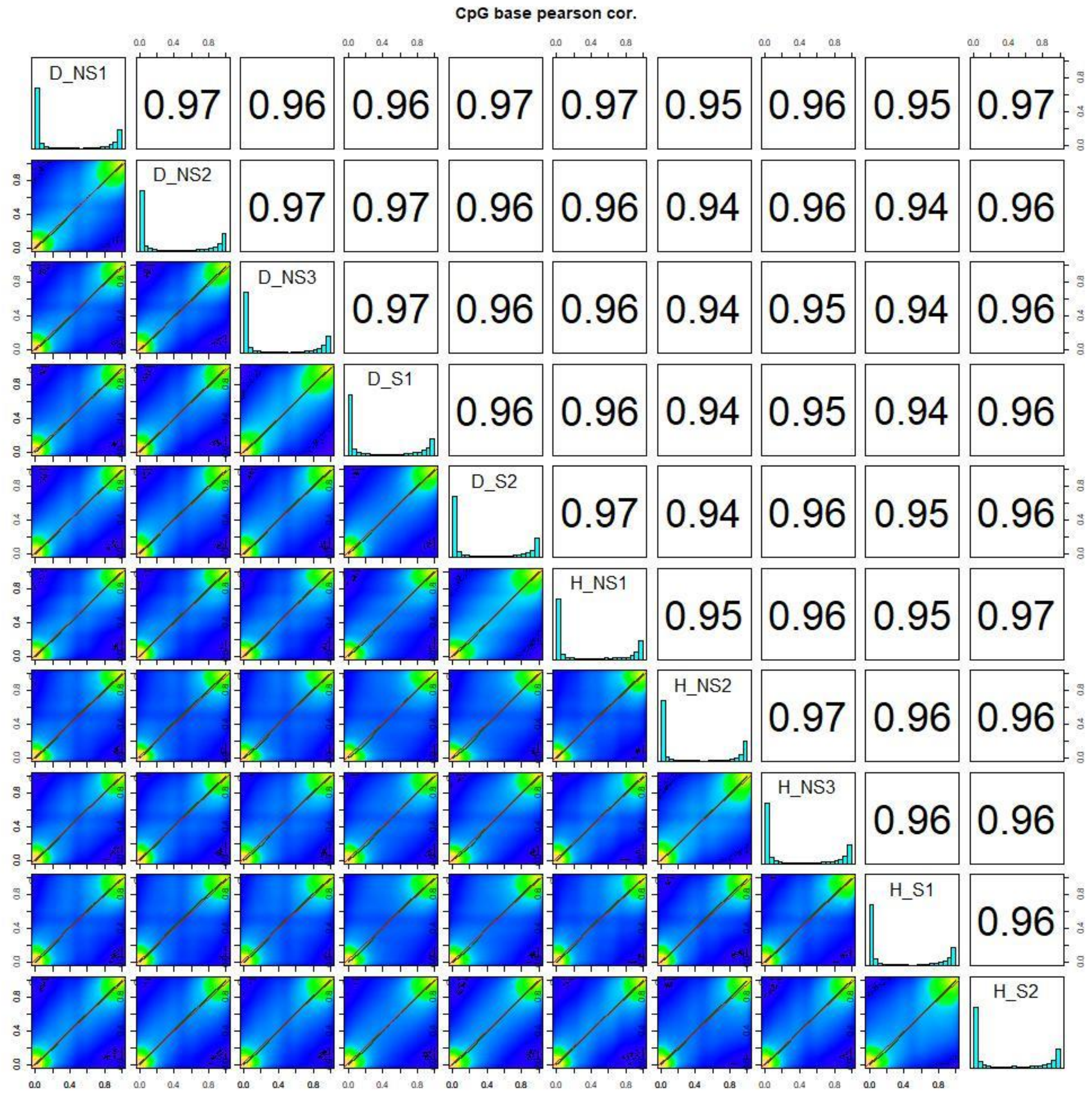

Figure S2. Pair-wise Pearson's correlation coefficients between different groups

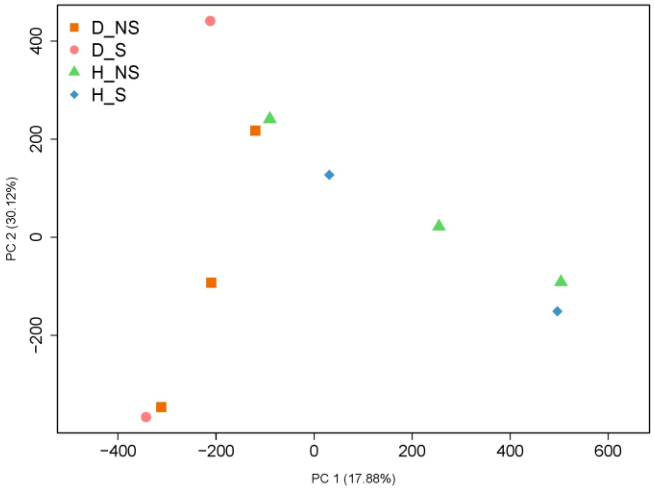

Figure S3. Principal Component Analysis (PCA) including all samples which demonstrates separation of the diseased and healthy samples.

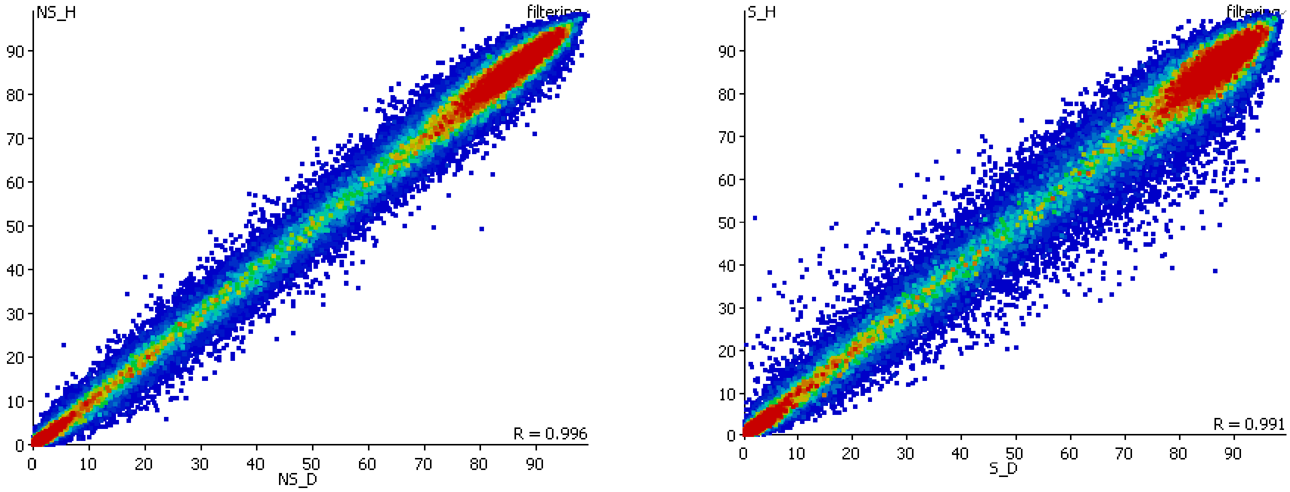

Figure S4 Scatter plots shown the correlation between healthy and diseased tissue in surgical and non-surgical group and the variation between the surgical replicates.

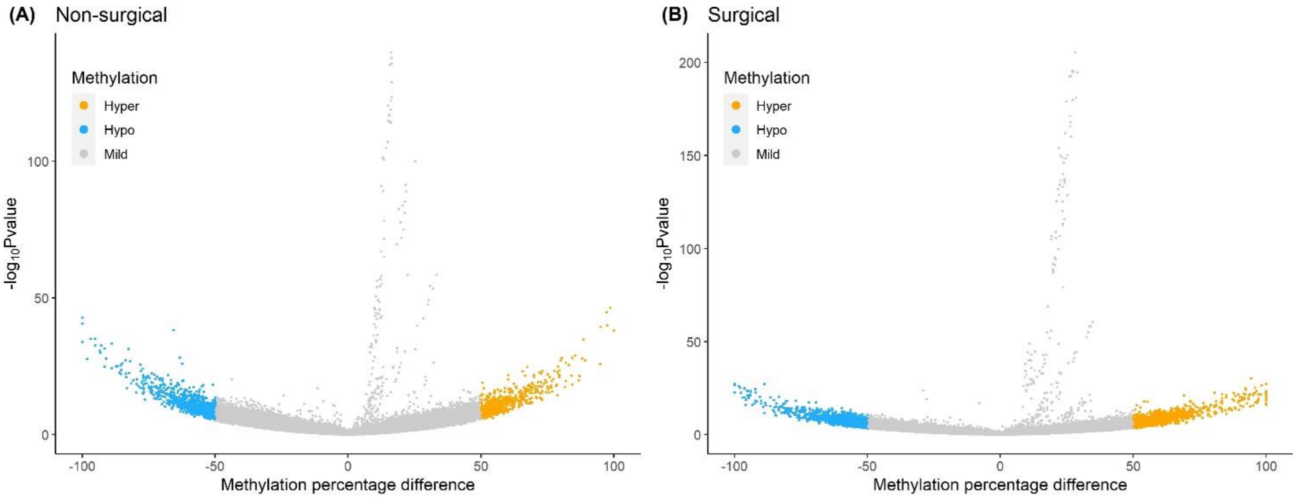

Figure S5 Volcano plots show the mean difference between diseased and healthy patient samples in non-surgical and surgical cohorts. Yellow dots represent the hypermethylated bases, and the blue dots represent the hypomethylated bases.

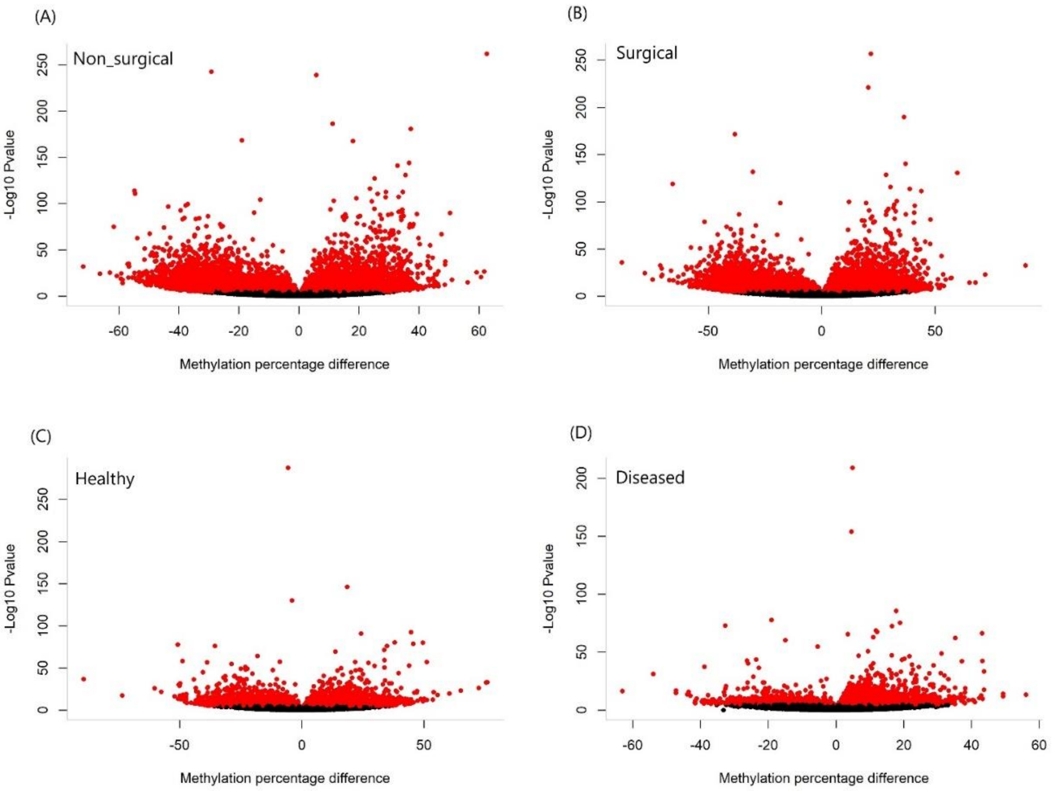

Figure S6 (A) & (B) Volcano plots show the mean difference between diseased and healthy patient samples in non-surgical and surgical cohorts. (C) & (D) show the mean difference between non-surgical patient samples and surgical patient samples in healthy and diseased.

A

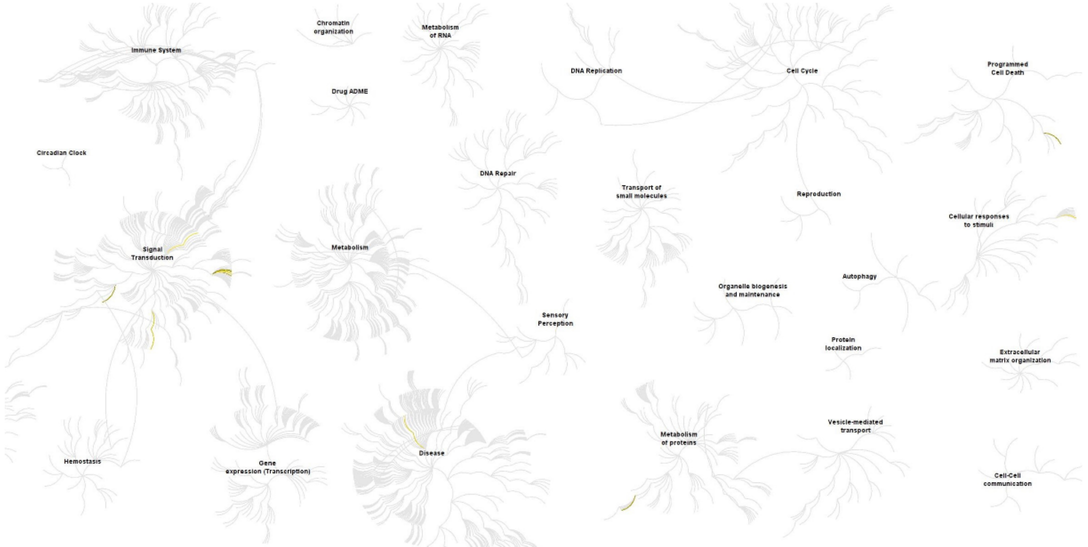

Pathway identifier	Pathway name	#Entities found	#Entities total	Entities ratio	Entities pValue	Entities FDR	#Reactions found	#Reactions total	Reactions ratio
R-HSA-186797	Signaling by PDGF	4	70	0.004558	1.06E-04	0.024959	24	31	0.002141
R-HSA-186763	Downstream signal transduction	3	37	0.002409	2.99E-04	0.035327	15	16	0.001105
R-HSA-9818030	NFE2L2 regulating tumorigenic genes	2	17	0.001107	0.001626374	0.126857	2	12	8.29E-04
R-HSA-9013026	RHOB GTPase cycle	3	75	0.004884	0.002273867	0.134158	3	6	4.14E-04
R-HSA-2219530	Constitutive Signaling by Aberrant PI3K in Cancer	3	96	0.006251	0.004532583	0.201327	1	2	1.38E-04
R-HSA-2219528	PI3K/AKT Signaling in Cancer	3	124	0.008074	0.009130356	0.201327	1	21	0.00145
R-HSA-6811558	PI5P, PP2A and IER3 Regulate PI3K/AKT Signaling	3	129	0.0084	0.010157082	0.201327	1	7	4.83E-04
R-HSA-199418	Negative regulation of the PI3K/AKT network	3	137	0.008921	0.011934714	0.201327	1	10	6.91E-04
R-HSA-8980692	RHOA GTPase cycle	3	154	0.010028	0.016274166	0.201327	3	6	4.14E-04
R-HSA-9013106	RHOC GTPase cycle	2	79	0.005144	0.03066401	0.201327	2	6	4.14E-04
R-HSA-72731	Recycling of eIF2:GDP	1	10	6.51E-04	0.033934064	0.201327	2	2	1.38E-04
R-HSA-416482	G alpha (12/13) signalling events	2	86	0.0056	0.035790828	0.201327	5	15	0.001036
R-HSA-351906	Apoptotic cleavage of cell adhesion proteins	1	11	7.16E-04	0.037264672	0.201327	1	10	6.91E-04
R-HSA-198203	PI3K/AKT activation	1	13	8.47E-04	0.043892127	0.201327	2	5	3.45E-04
R-HSA-9013423	RAC3 GTPase cycle	2	100	0.006512	0.046947174	0.201327	2	6	4.14E-04

B

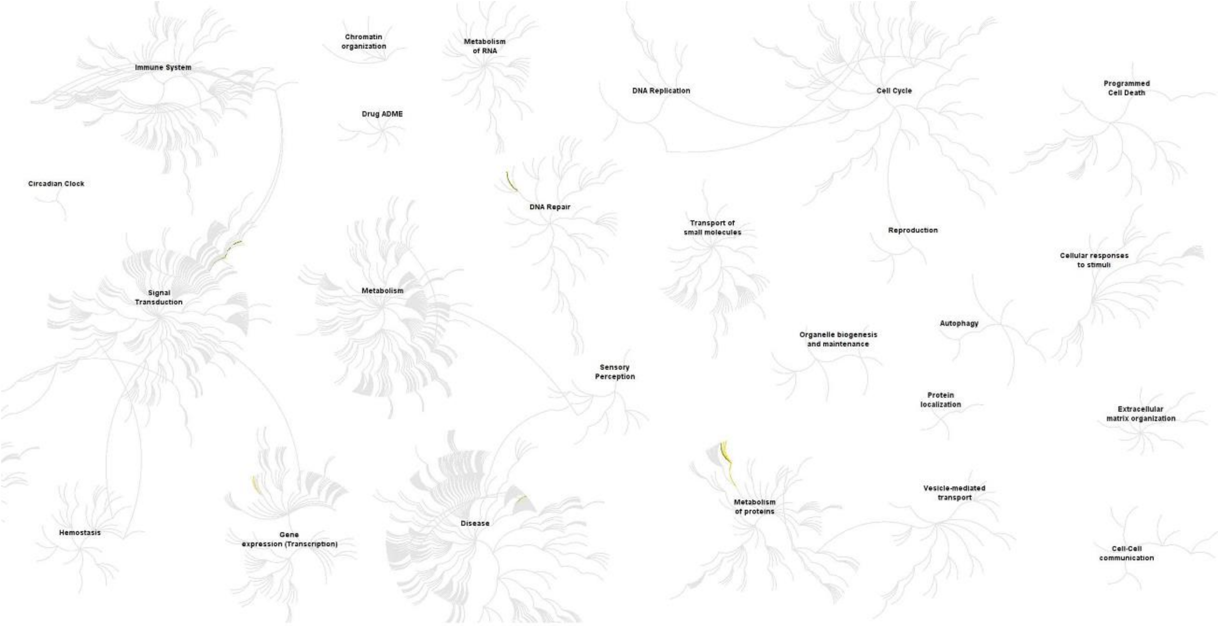

Pathway identifier	Pathway name	#Entities found	#Entities total	Entities ratio	Entities pValue	Entities FDR	#Reactions found	#Reactions total	Reactions ratio
R-HSA-3065679	SUMO is proteolytically processed	2	6	3.91E-04	8.74E-04	0.289361907	2	3	2.07E-04
R-HSA-3065676	SUMO is conjugated to E1 (UBA2:SAE1)	2	8	5.21E-04	0.001539159	0.289361907	2	3	2.07E-04
R-HSA-9022707	MECP2 regulates transcription factors	2	10	6.51E-04	0.002382804	0.297850524	2	8	5.52E-04
R-HSA-3215018	Processing and activation of SUMO	2	13	8.47E-04	0.003971554	0.373326048	5	9	6.21E-04
R-HSA-5619048	Defective SLC11A2 causes hypochromic microcytic anemia, with iron overload 1 (AHMIO1)	1	3	1.95E-04	0.021070157	0.387892662	1	1	6.91E-05
R-HSA-9025046	NTF3 activates NTRK2 (TRKB) signaling	1	4	2.60E-04	0.02799532	0.387892662	3	3	2.07E-04
R-HSA-9024909	BDNF activates NTRK2 (TRKB) signaling	1	4	2.60E-04	0.02799532	0.387892662	3	3	2.07E-04
R-HSA-9026357	NTF4 activates NTRK2 (TRKB) signaling	1	4	2.60E-04	0.02799532	0.387892662	3	3	2.07E-04
R-HSA-9026527	Activated NTRK2 signals through PLCG1	1	6	3.91E-04	0.041700351	0.387892662	3	3	2.07E-04
R-HSA-5696395	Formation of Incision Complex in GG-NER	2	47	0.003060494	0.044442457	0.387892662	2	10	6.91E-04
R-HSA-9032759	NTRK2 activates RAC1	1	7	4.56E-04	0.048480892	0.387892662	2	2	1.38E-04
R-HSA-3065678	SUMO is transferred from E1 to E2 (UBE2I, UBC9)	1	7	4.56E-04	0.048480892	0.387892662	1	3	2.07E-04

Figure S7 Reactome Pathway Analysis of differentially methylated genes in Non-Surgical (A) and Surgical (B) Groups. Overrepresented pathways are depicted by gold lines in the analysis. The tables showcase details of the top pathways with a p-value < 0.05.
